# P-1692. MRSA Antimicrobial Resistance Profiling: A Comparative Study of Database Performance and Correlation with Antimicrobial Susceptibility Testing Results

**DOI:** 10.1093/ofid/ofaf695.1866

**Published:** 2026-01-11

**Authors:** Norihito Kaku, Yasuhide Kawamoto, Fujiko Mitsumoto-Kaseida, Takahiro Takazono, Kosuke Kosai, Hiroo Hasegawa, Koichi Izumikawa, Hiroshi Mukae, Katsunori Yanagihara

**Affiliations:** Nagasaki University Hospital, Nagasaki, Nagasaki, Japan; Nagasaki University Hospital, Nagasaki, Nagasaki, Japan; Nagasaki University Graduate School of Biomedical Sciences, Nagasaki, Nagasaki, Japan; Nagasaki University Graduate School of Biomedical Sciences, Nagasaki, Nagasaki, Japan; Nagasaki University, Nagasaki, Nagasaki, Japan; Nagasaki University, Nagasaki, Nagasaki, Japan; Nagasaki University, Nagasaki, Nagasaki, Japan; Nagasaki University, Nagasaki, Nagasaki, Japan; Nagasaki University, Nagasaki, Nagasaki, Japan

## Abstract

**Background:**

Whole-genome sequencing (WGS) has been increasingly used for antimicrobial resistance (AMR) surveillance and molecular epidemiology. However, variability in AMR gene detection across databases may impact clinical interpretation. This study compared three AMR gene databases using WGS data on methicillin-resistant *Staphylococcus aureus* (MRSA) and assessed their correlation with antimicrobial susceptibility testing (AST) results.

**Figure:**
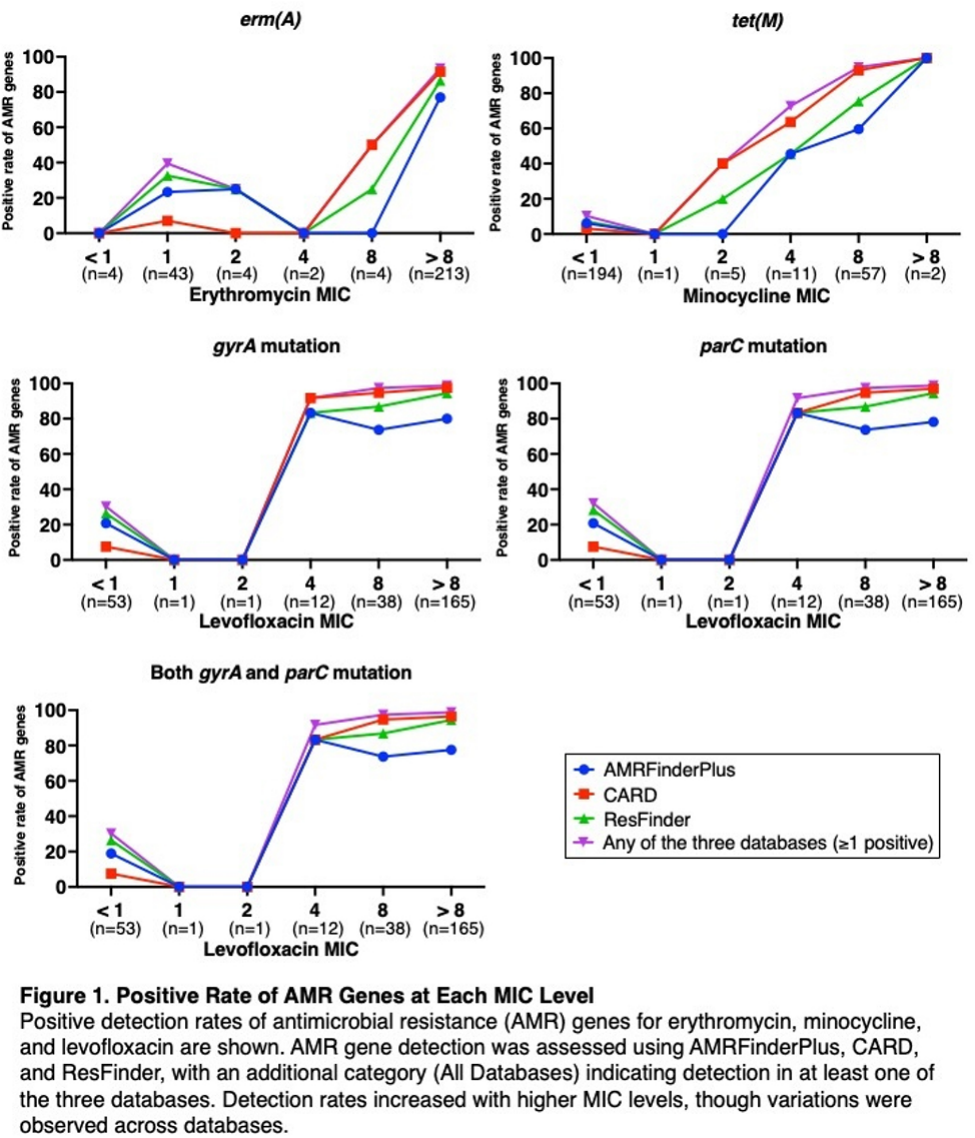

**Figure:**
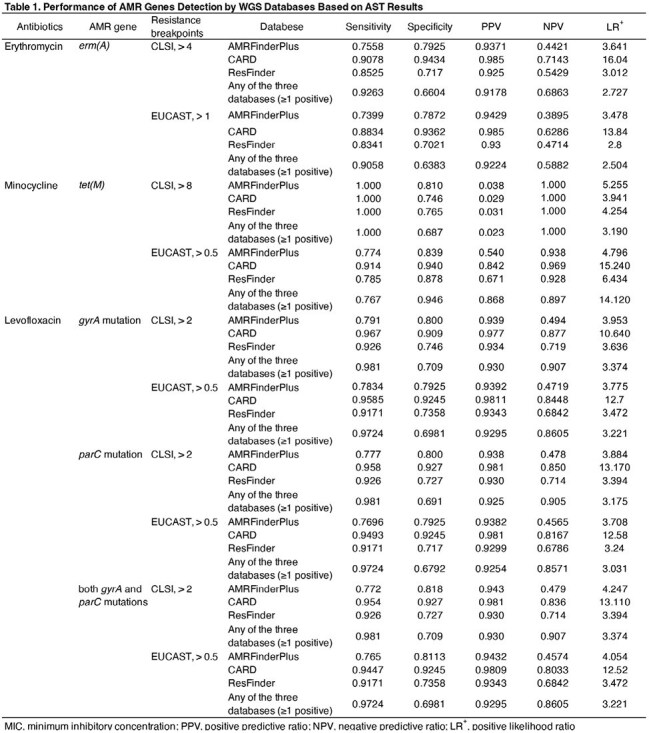

**Methods:**

We analyzed WGS data from 270 MRSA bloodstream infection isolates collected through nationwide surveillance in Japan. AMR genes were identified using AMRFinderPlus, CARD, and ResFinder. AST for erythromycin, minocycline, and levofloxacin was performed by broth microdilution, applying breakpoints defined by CLSI and EUCAST guidelines. Statistical analyses were conducted using GraphPad Prism 10.

**Results:**

The numbers of AMR genes detected by AMRFinderPlus, CARD, and ResFinder were 48, 53, and 42, respectively. The average number of AMR genes detected per strain was 8.3 ± 0.2 in AMRFinderPlus, 18.6 ± 0.1 in CARD, and 7.1 ± 0.2 in ResFinder. Despite all isolates being MRSA, *mecA* detection rates varied among databases, with detection rates of 82.2%, 98.1%, and 100% by AMRFinderPlus, CARD, and ResFinder, respectively.

Figure 1 shows the positive detection rates of AMR genes for erythromycin, minocycline, and levofloxacin at different MIC levels. The detection rates increased with higher MIC levels. The performance of WGS-based AMR gene detection in predicting AST results is summarized in Table 1. Sensitivity varied across antimicrobial classes and databases, with CARD demonstrating the highest sensitivity overall. LR⁺ values indicated that CARD had the strongest diagnostic accuracy for predicting AST-confirmed resistance. Sensitivity increased when considering resistance gene detection in at least one of the three databases, but this was accompanied by a decrease in the positive likelihood ratio (LR⁺).

**Conclusion:**

CARD demonstrated high sensitivity for erythromycin, minocycline, and levofloxacin. However, it did not detect *mecA* in a few isolates, raising concerns about MRSA identification. Integrating WGS-based AMR gene detection into clinical practice will require ongoing validation to ensure accuracy and reliability.

**Disclosures:**

All Authors: No reported disclosures

